# Aberrant R-loop-induced replication stress in MED12-mutant uterine fibroids

**DOI:** 10.1038/s41598-022-10188-x

**Published:** 2022-04-13

**Authors:** Sribalasubashini Muralimanoharan, Ross Shamby, Nicholas Stansbury, Robert Schenken, Barbara de la Pena Avalos, Samin Javanmardi, Eloise Dray, Patrick Sung, Thomas G. Boyer

**Affiliations:** 1 Department of Molecular Medicine, UT Health San Antonio, STRF, 8210 Floyd Curl Drive, Mail Code 8257, San Antonio, TX 78229-3900 USA; 2 Department of Obstetrics and Gynecology, UT Health San Antonio, San Antonio, TX USA; 3 Department of Biochemistry and Structural Biology, UT Health San Antonio, San Antonio, TX USA

**Keywords:** Cancer, Molecular medicine

## Abstract

Uterine fibroid (UF) driver mutations in Mediator complex subunit 12 (MED12) trigger genomic instability and tumor development through unknown mechanisms. Herein, we show that MED12 mutations trigger aberrant R-loop-induced replication stress, suggesting a possible route to genomic instability and a novel therapeutic vulnerability in this dominant UF subclass. Immunohistochemical analyses of patient-matched tissue samples revealed that MED12 mutation-positive UFs, compared to MED12 mutation-negative UFs and myometrium, exhibited significantly higher levels of R-loops and activated markers of Ataxia Telangiectasia and Rad3-related (ATR) kinase-dependent replication stress signaling in situ. Single molecule DNA fiber analysis revealed that primary cells from MED12 mutation-positive UFs, compared to those from patient-matched MED12 mutation-negative UFs and myometrium, exhibited defects in replication fork dynamics, including reduced fork speeds, increased and decreased numbers of stalled and restarted forks, respectively, and increased asymmetrical bidirectional forks. Notably, these phenotypes were recapitulated and functionally linked in cultured uterine smooth muscle cells following chemical inhibition of Mediator-associated CDK8/19 kinase activity that is known to be disrupted by UF driver mutations in MED12. Thus, Mediator kinase inhibition triggered enhanced R-loop formation and replication stress leading to an S-phase cell cycle delay, phenotypes that were rescued by overexpression of the R-loop resolving enzyme RNaseH. Altogether, these findings reveal MED12-mutant UFs to be uniquely characterized by aberrant R-loop induced replication stress, suggesting a possible basis for genomic instability and new avenues for therapeutic intervention that involve the replication stress phenotype in this dominant UF subtype.

## Introduction

Uterine fibroids (UFs; leiomyomas) are benign monoclonal neoplasms of the myometrium (MM) and represent the most frequent noncutaneous tumors in women worldwide^1^. Although benign, these tumors are nonetheless associated with significant morbidity; they are the primary indicator of hysterectomy, and a major source of gynecologic and reproductive dysfunction, ranging from menorrhagia and pelvic pain to infertility, recurrent miscarriage, and pre-term labor^2,3^. Accordingly, the annual US health care costs associated with UFs have been estimated at $34.9 billion^4^, rendering UFs a significant public health and financial burden.

Treatment options for UFs include pharmacologic or surgical intervention strategies designed, respectively, to mitigate clinical symptoms in the short-term or provide long-term remedial benefit^5^. Current pharmacological treatment strategies aim to lower circulating estrogen levels sufficiently to reduce hormone-dependent tumor growth while minimizing adverse hypoestrogenic effects systemically. For example, add-back therapies involving gonadotrophin-releasing hormone (GnRH) antagonists in combination with estrogen and progestin have recently been shown effective in reducing heavy menstrual bleeding and pain associated with UFs^6,7^. Nonetheless, treatment cessation is typically accompanied by symptom return and add-back therapy limits tumor regression, minimizing treatment impact on symptoms elicited by tumor bulk. Accordingly, alternative treatment options with superior tumor reductive properties are needed, and deeper insight regarding tumor etiology will be key to develop such agents.

In this regard, the underlying genetic drivers dominantly responsible for UF formation have now been largely identified. The most prevalent among these, accounting for ~ 70% of UFs, are somatic mutations in the gene encoding the RNA polymerase II transcriptional Mediator subunit MED12^8,9^. Mediator is an evolutionarily conserved multi-protein interface between signal-activated DNA-binding transcription factors and RNA polymerase II^10^. In this capacity, Mediator serves to channels regulatory signals from activator and repressor proteins to affect changes in gene expression programs that control diverse physiological processes, including cell growth and homeostasis, development and differentiation^10^. Within Mediator, MED12, along with MED13, CCNC (Cyclin C), and CDK8 (or its paralog CDK19) comprise a 4-subunit “kinase” module that variably associates with a 26 subunit Mediator core^11^. Notably, we and others have shown that MED12 activates CCNC-dependent CDK8 within Mediator and, further, that CDK8 kinase activity is required for nuclear transduction of signals instigated by multiple oncogenic pathways with established links to MED12 and UF biology, including the WNT/β-catenin, TGF-β, and other signaling axes^10,12–15^.

Regarding UF-linked alterations in MED12, all lie within exons 1 or 2 and most are missense mutations, with a smaller proportion corresponding to deletions, insertions, or splice site variants that nonetheless retain the MED12 open reading frame^9,13^. In addition to their high-frequency occurrence, the fact that MED12 mutations are predominantly allelically expressed in human UFs initially suggested a causative role in tumor formation^9,13,16^. More recently, this expectation was confirmed in a genetic mouse model, wherein uterine-specific expression of an orthologous *Med12* mutant allele most commonly found in human tumors (c. 131G > A; p.G44D) was shown sufficient to trigger UF formation in mice^17^. Notably, UF tumors arising in MED12-mutant mice were characterized by chromosomal aberrations, many with syntenic counterparts on human chromosomes, including 1p, 1q, 2q, 6p21, and 18p, known to be rearranged in human UFs^17^. Furthermore, prior studies have revealed that 60% of UFs with 6p21 rearrangements carried MED12 mutations^18,19^. Altogether, these observations suggest that MED12 mutations are precursors to chromosomal rearrangements that alter genome integrity and drive tumor progression. Nonetheless, the underlying mechanism by which MED12 mutations trigger genomic instability and tumor formation remains to be established.

In this regard, we previously showed that UF-linked mutations in MED12 disrupt its ability to activate CDK8 in Mediator, thus revealing Mediator kinase disruption to be an immediate downstream biochemical defect arising from these genetic alterations^13–15^. Mechanistically, this occurs through alteration of the CDK8 activation (T)-loop, since we and others recently showed that MED12 exon 2-encoded residues comprise a CDK8 “activation helix” that lies adjacent to the CDK8 T-loop in a MED12-CCNC-CDK8 ternary complex and, further, that UF-linked MED12 mutations promote a T-loop conformation incompatible with substrate binding and phosphorylation^11,20^. Notably, the Mediator kinase module has previously been implicated in the maintenance of genomic integrity through control of R-loop biology^21,22^, suggesting a possible basis to explain UF formation, at least in part, through MED12 mutation-induced R-loop accrual and genomic instability.

Co-transcriptional R-loops, byproducts arising through hybridization of nascent RNA with the template DNA strand, constitute an established threat to genomic integrity^23^. At physiological levels, R-loops play important gene regulatory roles, including immunoglobulin class switch recombination, promoter chromatin patterning, and transcription termination^24^. However, if left to accumulate to pathological levels through excessive production and/or inefficient processing, R-loops can form barriers to the replisome and educe replication delay (replication stress) and fork collapse, leading to DSBs^25,26^. Intriguingly, R-loop domains are associated with two epigenetic modifications, including histone 3 serine 10 phosphorylation (H3S10p) and H3 lysine 9 di-methylation (H3K9me2), that we and others have shown to be regulated by MED12 and Mediator kinase activity in mammalian cells^27–30^. Moreover, prior work in yeast has shown that genetic disruption of the Mediator kinase module triggers an increase in R-loop-induced genomic instability, suggesting a physiological role for the Mediator kinase module in R-loop suppression and maintenance of genomic integrity^22^. Because UF driver mutations in MED12 disrupt Mediator kinase activity that is linked to R-loop suppression and maintenance of genomic integrity, we investigated R-loop metabolism, replication stress, and their functional relationship in UFs and cultured uterine smooth muscle cells as a function of MED12 mutations and downstream Mediator kinase disruption.

## Results

### MED12-mutant UFs are characterized by aberrant R-loop accrual and replication stress

**Figure 1:**
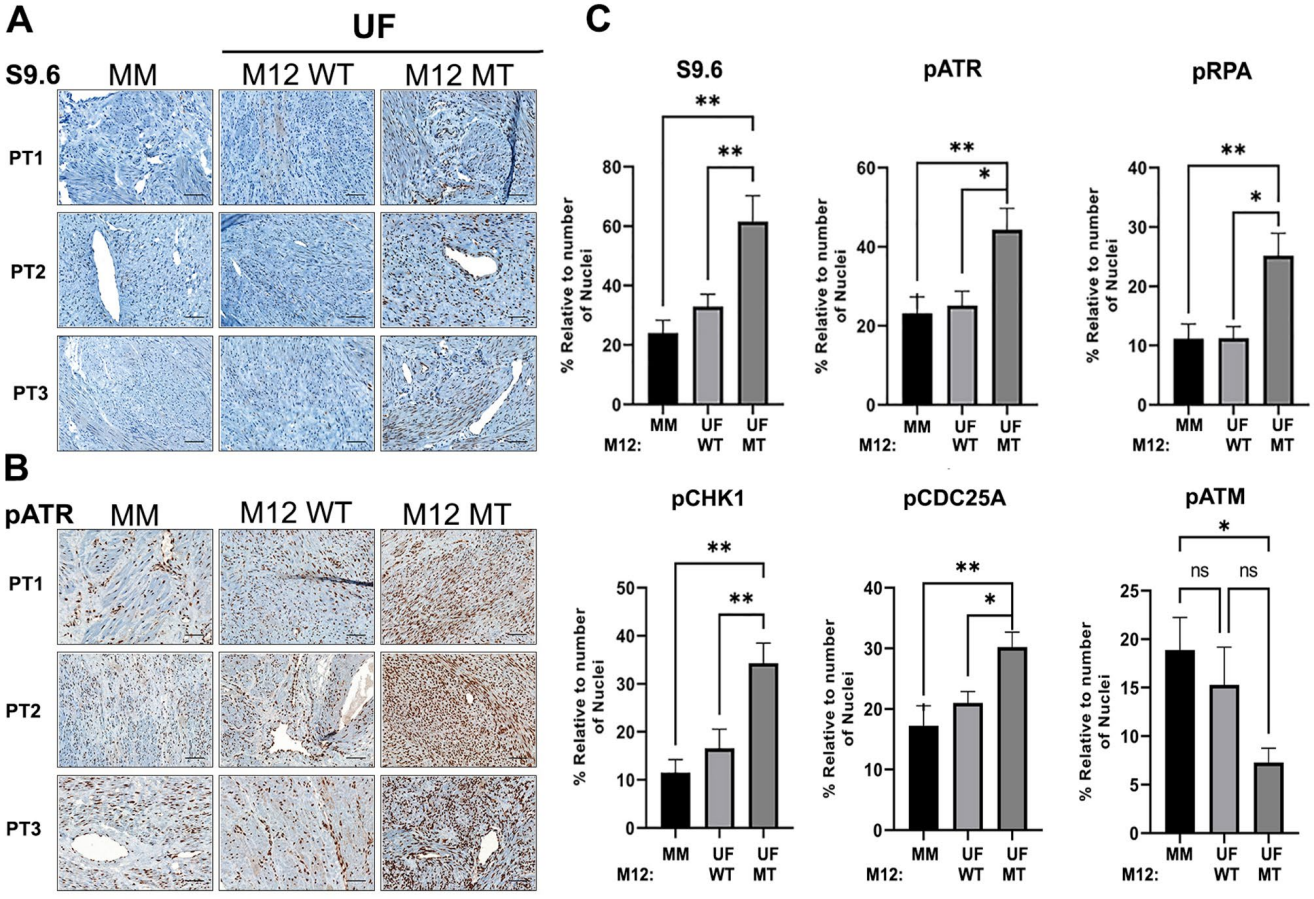
MED12-mutant UFs are characterized by aberrant R-loop accrual and activated replication stress signaling. (**A** and **B**) Representative IHC staining with (**A**) R-loop specific S9.6 and **(B**) pATR (Ser428) specific antibodies. Shown are images from 3 patient-matched tissue sample sets (PT1-PT3), each set comprising MM, MED12-wild-type (M12 WT) and MED12-mutant (M12 MT) UF tissue. (**C**) Quantified expression of S9.6 and phosphorylated (activated) replication stress signaling markers pATR (Ser428), pRPA (Ser33), pCHK1 (Ser317), pCDC25A (Ser124), and pATM (s1981). Data were quantified from IHC analysis of 10 patient-matched tissue sample sets. Stained sections were scanned using an Aperio ScanScope® CS system, and individual nuclei as well as nuclear and cytoplasmic marker localization were identified and quantified using Aperio ImageScope software. Signals were normalized to the number of nuclei in each section. Statistical significance was calculated using One-Way ANOVA followed by Tukey’s Post hoc test, **p ≤ 0.01; *p ≤ 0.05.

To determine if R-loops preferentially accumulate in MED12-mutant UFs, we performed immunohistochemical (IHC) analysis using the RNA–DNA hybrid-specific antibody S9.6 to probe tissue microarrays (TMAs) encompassing 10 patient-matched sample sets, each set comprising tissue from MM as well as one MED12-WT and one MED12-mutant UF tumor all derived from the same patient uterus. Strikingly, MED12-mutant UFs, compared to MM and MED12-WT UFs, were characterized by significantly enhanced S9.6 immunoreactivity that was completely abolished by pre-treatment of tissue arrays with RNaseH that degrades the RNA strand of the RNA–DNA hybrid (Fig. 1A and C; Supplemental Fig. S1). Thus, MED12-mutant UFs are uniquely characterized by aberrant accumulation of R-loops.

Because R-loop accrual can evoke replication stress^25,26^, we processed TMAs for IHC analysis with additional antibodies specific for established replication stress signaling proteins, including total and phosphorylated (activated) forms of the ssDNA-binding replication protein A (RPA), the master replication stress kinase Ataxia Telangiectasia and Rad3-related (ATR), and ATR signaling mediators CHK1 and CDC25A. Additionally, TMAs were probed for the activated form of the master DNA double-strand break (DSB) response kinase Ataxia Telangiectasia Mutated (ATM). This analysis revealed that MED12-mutant UFs, compared to MM and MED12-WT UFs, exhibited significantly higher levels of phosphorylated, but not bulk, stress signaling proteins tested, indicating that MED12-mutant UFs are characterized by activation of the ATR-CHK1 replication stress signaling axis (Fig. 1B and C; Supplemental Figs. S2 and S3). Notably, MED12-mutant UFs showed significantly reduced levels of activated ATM compared to MM (Fig. 1C; Supplemental Figs. S2 and S3), indicating that MED12-mutant UFs selectively activate the replication stress-induced ATR pathway, but not the DSB-induced ATM pathway.

**Figure 2:**
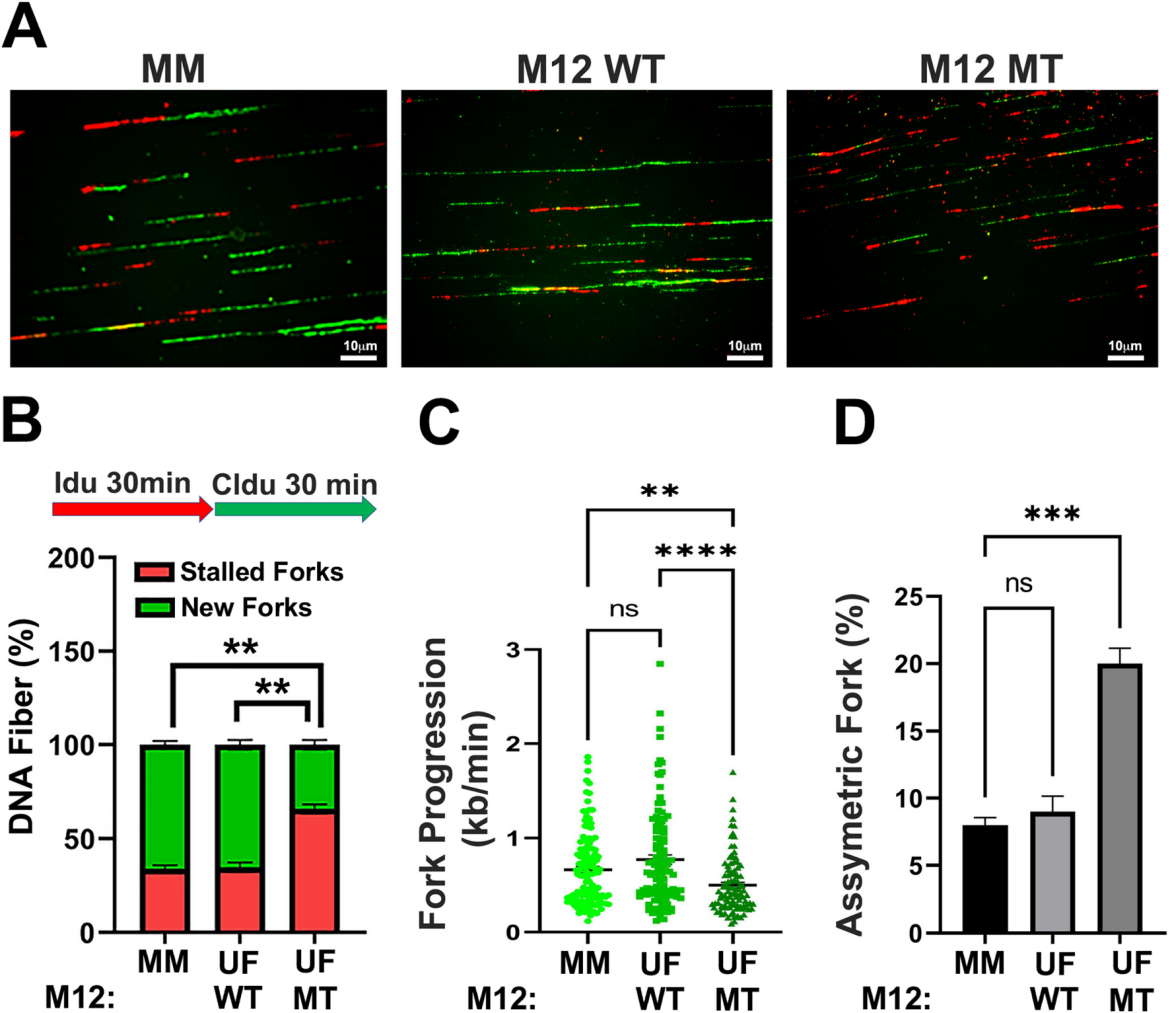
MED12-mutant UFs are characterized by replication stress. (**A**) Representative images of combed DNA fibers from primary MM and UF cells pulse-labeled with thymidine analogues IdU (red) and CldU (green). The MED12 mutation status of UF cells [MED12 WT (M12 WT) or MED12-mutant (M12 MT)] is indicated. (**B**) Top, pulse labeling scheme. Bottom, percentage of singly labeled fibers retaining only first (Idu) or second (CldU) label indicative of stalled or new forks, respectively. (**C**) Velocity of fork progression as determined from ldU tract lengths (kb/min). (**D**) Percentage of bidirectional asymmetric forks, represented as percentage of forks relative to total number of forks in the field. Statistical significance was calculated using One-Way ANOVA followed by Tukey’s Post hoc test, ****p ≤ 0.0001, ***p < 0.001; **p < 0.01. ns, not significant.

To determine if enhanced R-loop levels and activated ATR-CHK1 signaling in MED12-mutant UFs are associated with overt replication stress, we assessed replication fork dynamics in primary UF and MM cell cultures using single molecule DNA fiber analysis. To this end, single cell suspensions from patient-matched MM and UFs, including MED12 mutation-positive and MED12 mutation-negative tumors (Supplemental Fig. S4), were plated and processed for DNA fiber analysis within 3 days in order to ensure retention of MED12-mutant cells that are otherwise lost within the first several passages in culture^31^. Plated cells were pulse labeled with thymidine analogues IdU and CldU, and DNA fibers from lysed cells were combed and immunodetected using analogue-specific antibodies. First, we investigated replication fork stalling and new origin firing in primary MM and UF cells. We found that the number of stalled replication forks (DNA fibers containing only IdU tracts) was significantly higher, while the number of new replication origins (DNA fibers containing only CldU tracts) was significantly lower, in MED12-mutant UF cells compared to cells from MM or MED12-WT UFs (Fig. 2A and B). Next, we measured replication fork speed based on IdU labeling. We observed significantly shorter fork lengths in MED12-mutant UF cells compared to those from either MM or MED12-WT UF cells, neither of which significantly differed from the other (Fig. 2C). These findings indicate that UF driver mutations in MED12 reduce replication fork progression. Importantly, staining of combed DNA fibers with fluorescent antibody specific for single-stranded DNA confirmed fiber integrity, revealing that shortened singly labeled tracts observed in MED12-mutant cells derive from altered replication fork dynamics as opposed to broken DNA fibers arising during the combing process (Supplemental Fig. S5). Finally, in addition to fork slowing and stalling, bidirectional asymmetric forks are also associated with replication stress. These derive from asymmetrical fork progression through replication fork arrest. We found that MED12-mutant UF cells, compared to cells from MM or MED12-WT UFs, exhibited significantly more bidirectional asymmetric forks (Fig. 2D). Together, these results indicate a function for MED12 in the control of proper replication fork dynamics, one that is compromised by UF driver mutations in exon 2.

Because replication stress can lead to fork collapse and DSBs^25,26^, we investigated whether MED12-mutant UFs support elevated levels of DNA breaks. To this end, TMAs were additionally probed for markers of DSBs, including p53BP1 and γH2AX. Neither marker was specifically elevated in MED12-mutant UFs compared to MM or MED12-WT UFs (Supplemental Fig. S6), consistent with reduced activation of ATM kinase that we observed in MED12-mutant tumors (Fig. 1C). Altogether, these findings reveal that MED12-mutant UFs are uniquely characterized by aberrant R-loop accrual and activation of the ATR-CHK1 signaling axis in response to ongoing replication stress without extensive accumulation of DSBs, indicative of an intact S-phase checkpoint in this setting.

### Mediator kinase inhibition triggers R-loop-induced replication stress and activation of the S-phase checkpoint in uterine smooth muscle cells

Recently, we showed that UF driver mutations in MED12 disrupt CCNC-CDK8 activity in Mediator by altering CDK8 T-loop stabilization, implicating reduced Mediator kinase activity in their pathogenic sequelae^11,13–15^. To test this prediction, and further investigate the functional relationship between R-loops and replication stress, we asked whether direct inhibition of Mediator kinase activity could recapitulate the impact of mutant MED12 on R-loop accrual and replication stress in uterine smooth muscle cells (UtSMCs) otherwise carrying WT MED12. To this end, we treated UtSMCs with a highly specific chemical inhibitor of CDK8/19, CCT251545^32^, and monitored R-loop levels thereafter by immunocytochemical analysis using S9.6 antibody. Target engagement and Mediator kinase inhibition by CCT251545 under these experimental conditions was confirmed by analysis in parallel of INFγ-stimulated STAT1 phosphorylation on serine residue 727 (pSTAT1^SER727^), a validated biomarker of Mediator kinase activity in vivo^32,33^. This analysis revealed an inverse temporal relationship between Mediator kinase inhibition and R-loop formation. Thus, relative to DMSO-treated cells in which steady-state R-loop levels were essentially undetectable, R-loop accumulation in CCT251545-treated cells was first observed at 15 min following inhibitor treatment, when Mediator kinase activity began to decline, and peaked 2 h thereafter, when Mediator kinase activity was completely inhibited (Supplemental Fig. S7). This suggests that R-loops are caused by Mediator kinase inhibition.

**Figure 3:**
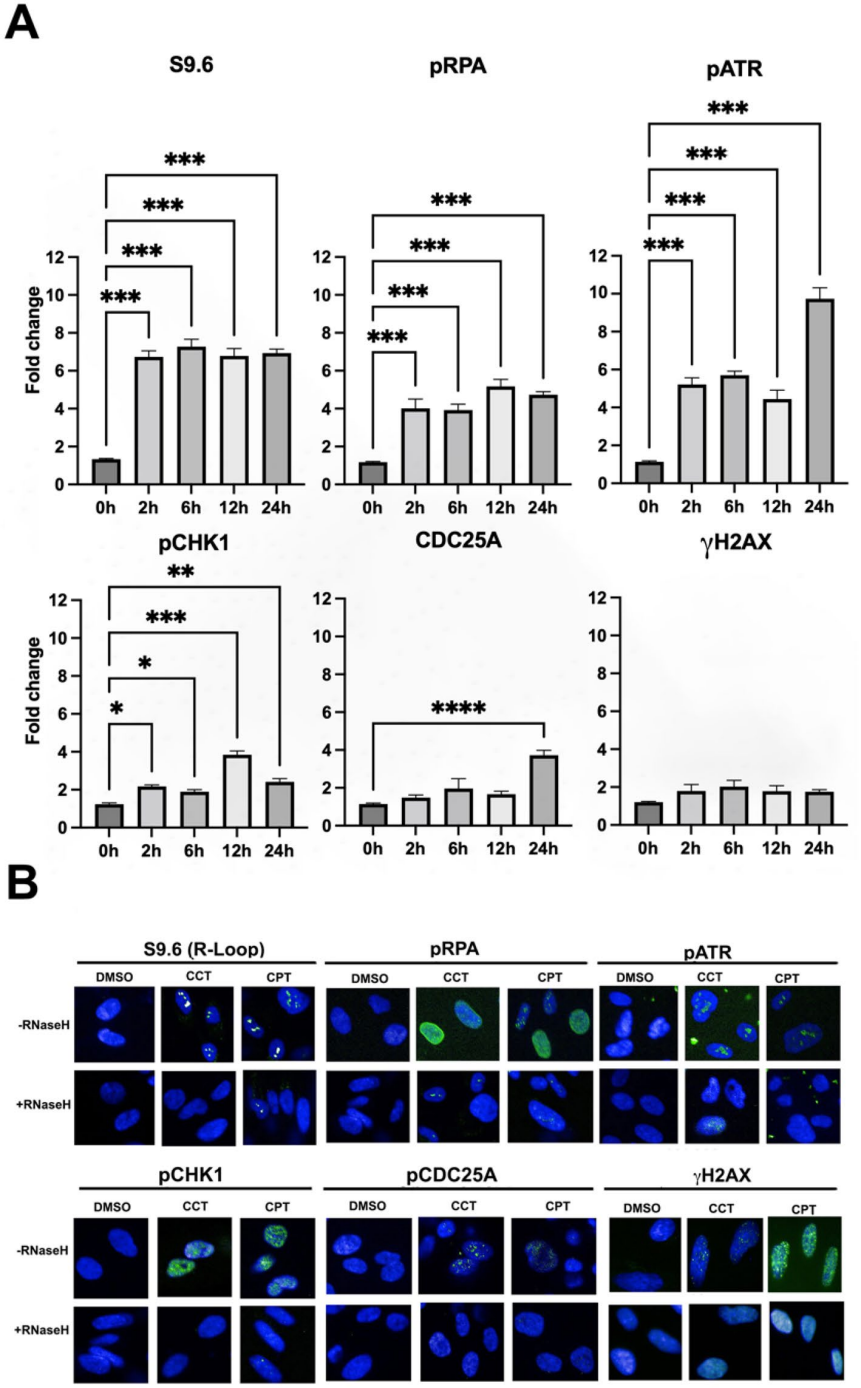
Mediator kinase inhibition triggers R-loop induced replication stress in UtSMCs**.** (**A**) Temporal kinetics of replication stress marker activation in UtSMCs following Mediator kinase inhibition. UtSMCs were treated with CCT251545 (100 nM) for the indicated time points prior to immunocytochemical analysis with antibodies specific for R-loops (S9.6), activated replication stress signaling markers [pRPA (Ser33), pATR (Ser428), pCHK1 (Ser317), pCDC25A (Ser124)] and the DNA damage marker γH2AX. Data are plotted as fold-change in antibody signal intensity relative to untreated cells (0 h). Statistical significance was calculated using One-Way ANOVA followed Tukey’s Post hoc test, ****p ≤ 0.0001, ***p < 0.001; **p < 0.01. and *p ≤ 0.05. (**B**) Representative images of data plotted in (B). Images were captured at the peak time points for each marker. A parallel experiment was performed in UtSMCs overexpressing RNaseH (+ RNaseH). Campothecin (CPT; 10 μM) was included as positive control for R-loop induced replication stress signaling.

**Figure 4:**
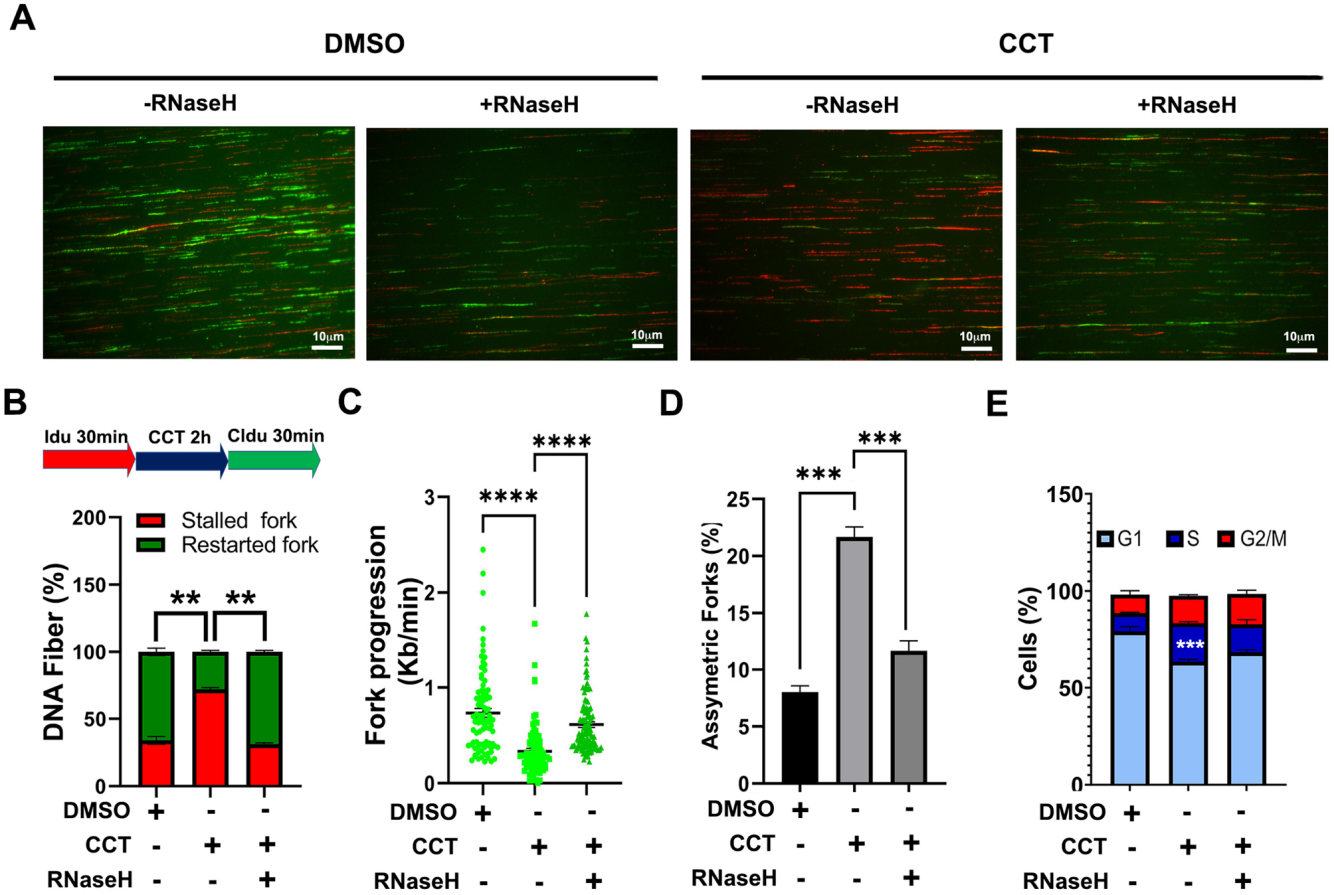
Mediator kinase inhibition alters replication fork dynamics and triggers S-phase delay in UtSM cells. (**A**) Representative images of combed DNA fibers from IdU/CldU pulse-labeled parental or RNAaseH overexpressing (−/ + RNaseH) UtSMCs cells treated with DMSO or CCT251545 (CCT; 100 nM). (**B**) Top, pulse labeling scheme. CCT251545 was added for 2 h between 30 min Idu (red) and CldU (green) pulses. Bottom, percentage of singly labeled fibers retaining only first (Idu) or second (CldU) label indicative of stalled or re-started forks, respectively. (**C**) Velocity of fork progression as determined from CldU tract lengths (kb/min). (**D**) Percentage of bidirectional asymmetric forks, represented as percentage of forks relative to total number of forks in the field. (**E**) Unsynchronized parental or RNaseH overexpressing (−/ + RNaseH) UtSMCs treated with DMSO or CCT251545 (CCT; 100 nM) for 3 days were stained with propidium iodide and analyzed by flow cytometry. Percentage of cells in each cell cycle phase is plotted. Statistical significance was calculated using One-Way ANOVA followed Tukey’s Post hoc test, ****p ≤ 0.0001, ***p < 0.001; **p < 0.01.

To determine if R-loops induced by Mediator kinase inhibition trigger replication stress, we monitored the activation kinetics of stress signaling markers and replication fork dynamics by immunocytochemistry and DNA fiber analysis, respectively, in CCT251545-treated UtSMCs cells. Indeed, we observed that Mediator kinase inhibition triggered activation of the ATR-CHK1 replication stress signaling axis (Fig. 3A and B). Notably, we also found that Mediator kinase inhibition led to increased and decreased numbers of stalled and restarted replication forks, respectively (Fig. 4A and B), reduced replicative DNA fiber lengths (Fig. 4C), and increased numbers of bidirectional asymmetric forks (Fig. 4D). Importantly, these phenotypes were effectively reversed in Mediator kinase-inhibited UtSMCs ectopically overexpressing catalytically active RNaseH, indicating that replication stress triggered upon Mediator kinase inhibition is R-loop dependent (Fig. 3B and Fig. 4A-D; Supplemental Fig. S8A).

To determine if R-loop-dependent replication stress triggered by Mediator kinase inhibition leads to enhanced DNA damage, we performed immunocytochemical analysis on control and CCT251545-treated UtSMCs cells using antibody specific for the DSB marker γH2AX. Consistent with our observations in MED12-mutant UF tumors (Supplemental Fig. S6), γH2AX levels were not appreciably increased in Mediator kinase-inhibited UtSMCs despite ongoing replication stress (Fig. 3A and B), indicative of an intact checkpoint response to altered replication fork dynamics. Because we observed that R-loop-induced replication stress activates the ATR-CHK1 pathway that is known to enforce an intra-S-phase checkpoint^34^, we monitored the proliferative capacities and cell cycle profiles of Mediator kinase-inhibited UtSMCs without or with ectopic overexpression of RNAaseH. Compared to DMSO-treated UtSMCs, CDK8 inhibitor-treated cells exhibited a proliferative defect accompanied by an S-phase cell cycle delay, phenotypes that were significantly reversed by overexpression of RNaseH (Fig. 4E; Supplemental Figs. S8 and S9). Altogether, these results indicate that Mediator kinase inhibition triggers R-loop induced replication stress leading to activation of an ATR-dependent S-phase checkpoint in UtSMCs.

## Discussion

Although UF-linked MED12 mutations are proven tumor drivers, the molecular basis for their tumorigenic activity is nonetheless poorly understood. Prior genetic studies have suggested that MED12 mutations may be precursors to genomic alterations that promote tumorigenesis^17–19^, but how these mutations trigger genomic instability remains to be fully elucidated. Herein, we show that MED12 mutation-positive UFs are characterized by aberrant R-loop accrual and altered replication fork dynamics indicative of replication stress. Moreover, we found that these phenotypes could be recapitulated and functionally linked in UtSMCs by targeted disruption of Mediator kinase activity corresponding to the principal biochemical defect arising from UF driver mutations in MED12. Notably, despite ongoing replication stress, extensive DNA damage was not observed in MED12-mutant tumors and Mediator kinase-inhibited cells, indicative of an intact checkpoint, which was further confirmed by evidence of activated ATR-CHK1 signaling and delayed S-phase cell cycle kinetics. Consistent with low-level DNA damage observed in MED12-mutant and Mediator kinase-inhibited cells, the 2–threefold reduction in replication fork speeds measured in these cells compared to their normal counterparts reflects moderate replication stress as opposed to severe replication stress that instead leads to collapse of completely stalled replication forks into DSBs^34^. This phenotype could explain the inherent, yet nonetheless comparatively limited, genomic instability in benign UF tumors compared to malignant tumors, including highly aggressive uterine leiomyosarcomas. Furthermore, in contrast to severe replication stress that limits mitotic entry of damaged DNA in checkpoint-proficient cells, mild replication stress may escape such surveillance, resulting in mitotic entry of under-replicated or unresolved DNA structures that are otherwise susceptible to mitotic breakage and chromosomal aberrations^35–38^. Accordingly, the low-level chronic replication stress that we observe in MED12-mutant UFs could pose a threat to genomic integrity through mechanisms involving mitotic checkpoint evasion or bypass.

On the other hand, replication stress has also been linked with the onset of cellular senescence, which has been observed in up to 50% of UFs, as well as other benign slow-growing tumor types^39,40^. In fact, the early growth arrest phenotype associated with cellular senescence has been proposed to serve as an effective barrier to malignant transformation of UFs and certain premalignant tumor types, while later acquisition of a senescence-associated secretory phenotype could otherwise serve to promote tumor progression due to proinflammatory and immunosuppressive properties of the senescent secretome^40,41^. Future studies will be required to clarify the biological consequences of chronic replication stress in MED12-mutant UFs and their specific contribution to tumorigenesis.

The moderate replication stress phenotype identified herein for MED12-mutant UFs suggests a possible therapeutic vulnerability for this dominant UF subclass. In this regard, inhibitors of the ATR axis, including ATR, CHK1, and WEE1, have been used to effectively target tumor cells with increased replication stress^42,43^. However, the clinical utility of such drugs is often constrained in tumor cells with high levels of chemotherapeutic drug-induced replications stress, where combination therapy triggers excessive origin firing and RPA exhaustion in S-phase arrested cells, leading to DNA damage and cell death in both tumor and normal tissues^44^. By contrast, tumor cells with moderate replication stress that feature reserve RPA and delayed S-phase progression, can nonetheless be pushed to RPA exhaustion, S-phase arrest, and DNA damage-induced cell death by CHK1 inhibitors that activate late origin firing^44^. Importantly, this occurs at drug doses with little collateral impact on normal tissue, effectively widening the therapeutic window available for treatment. Accordingly, CHK1 inhibitors may prove clinically effective in MED12-mutant UFs characterized by chronic low-level replication stress.

Recently, the Mediator kinase subunits CDK8 and CCNC have been implicated in mechanisms of ATR inhibitor (ATRi) resistance, supporting the link identified herein between the Mediator kinase module and replication stress signaling. These reports, based largely on loss of function CRISPR-knockout and RNAi-mediated screening and validation approaches, revealed that ATRi resistance conferred by loss of CCNC or CDK8 could be attributed to reduced levels of transcription-dependent replication stress^21,45^. However, as shown specifically for CDK8, as well as other kinases that function in multiprotein complexes, reduced protein abundance and reduced enzymatic activity are not phenotypically equivalent, indicating that kinase-independent (i.e., scaffolding) functions contribute to overall CDK8 function in cells^46–49^. Our findings herein distinguish between these scenarios and identify a critical role for Mediator kinase activity, known to be disrupted by MED12 UF driver mutations, in suppression of R-loop induced replication stress.

Presently, the mechanistic basis which MED12 mutations and Mediator kinase disruption trigger aberrant R-loop accrual and downstream replication stress is unclear. Because Mediator kinase is implicated in the control of RNA Polymerase II pausing and elongation^10^, it is conceivable that impaired Mediator kinase activity leads to stalled polymerases, a known source of R-loops, that in turn elicit transcription-replication conflicts and ensuing replication stress^23,50^. Alternatively, or additionally, Mediator kinase disruption could more directly impact replication dynamics, a possibility supported by recent studies that invoke a direct role for CDK8 and MED12, respectively, in replication origin firing and fork stability^45,51^. In this regard, among a catalog of high-confidence Mediator kinase substrates that we recently identified in myometrial stem cells, we note that transcription and replication associated proteins were prominently represented, consistent with either of these two possibilities^33^. Further studies will be required to determine the precise mechanism by which Mediator kinase disruption as a consequence of MED12 mutations triggers R-loop induced replication stress in UFs. In summary, we found that MED12-mutant UFs are uniquely characterized by aberrant R-loop induced replication stress, suggesting a possible route to genomic instability and new avenues for therapeutic intervention that involve the replication stress phenotype in this dominant UF subclass.

## Materials and methods

### Patients and samples

This study was approved by the institutional review board of UT Health San Antonio. MM and UF samples were collected from patients (n = 12) providing informed consent and undergoing hysterectomy. MM and UF tissues from 10 patients were described previously^13^ and used for tissue microarray (TMA) generation, while MM and UF tissues from 2 additional patients were used for primary cell cultures. Demographic and clinicopathological information for all 12 patients in the current study is provided in Supplementary Table S1.

### Cell culture

Immortalized uterine smooth muscle cells (UtSMCs) have been described^52^ and were cultured in DMEM/F12 (GIBCO) with 10% fetal bovine serum and 1% antibiotic–antimycotic (Invitrogen) at 5% CO2. Primary MM and UF cells were similarly cultured in DMEM/F12 (GIBCO) with 10% fetal bovine serum and 1% antibiotic–antimycotic (Invitrogen) at 5% CO2 and processed for DNA fiber analysis (see below) within 3 days of initial plating.

### Tissue micro array and immunohistochemistry

Matched MM and UF (MED12 mutation-negative and MED12 mutation-positive) tissues from 10 patients were used for TMA generation. A board-certified pathologist (Dr. Robert Reddick, UT Health San Antonio) reviewed the hematoxylin–eosin-stained sections cut from formalin-fixed, paraffin-embedded tissue blocks for all patients and identified regions for creation of TMAs that included fibroids and normal adjacent myometrium. The TMAs were constructed as previously described^53^. Tissue cores with a diameter of 2 mm were punched from each specimen in triplicate and arrayed into a recipient paraffin block. The template used to produce the TMAs was designed using randomized samples within the template to avoid artifacts caused by technical problems. TMA slides consist of 30 tissue sections including 10 individual patient-matched sample sets, each set comprising MM, as well as MED12 mutation-negative UF and MED12 mutation-positive UF tissues, all derived from the same patient uterus. Orientation markers were added to the template for correct alignment.

Immunohistochemistry was performed on the TMA slides using a Ventana XT Immunostainer with I-View DAB detection, which uses an avidin–biotin detection system. Secondary antibody alone was used on off cuts of tissue microarray slides as a negative control. Slides were stained with antibodies specific for proteins of interest, including S9.6 (AB-2687463; Kerafast), and Mybiosource Antibodies pRPA (MBS2534546), RPA (MBS2534546), pATR (MBS9608121), ATR (MBS9612974), pCHK1 (MBS9600779), CHK1 (MBS767885), pCD25A (MBS9201824), CDC25A (MBS629584), γH2AX (MBS9412572), pATM (M128339), ATM (MB2535445), p53BP1 (MBS9413890). Slides were scanned using the Aperio ScanScope® CS system (Leica Microsystems GmbH, Wetzlar, Germany) with a 40 × objective lens. The histologic parameters were evaluated using Aperio ImageScope software (version 12.3.2.8013; Leica Microsystems). Individual nuclei and nuclear and cytoplasmic localization of protein of interest were identified using the default algorithm built into the ImageScope software. The quantified intensity was normalized to the number of nuclei of the sections in the TMA slides.

### Generation of stable RNaseH-expressing uterine smooth muscle cell line

A lentiviral V5-tagged WT RNaseH1-expressing plasmid was generated from ppyCAG_RNaseH1-WT (addgene #111,906)^54^ and LVR-1003-MCS-PGK-BSD (Cellomics Technology). Briefly, the coding sequence (CDS) for C-terminally V5-tagged WT RNase H1 (carrying a nuclear localization signal) was PCR-amplified from ppyCAG_RNaseH1-WT using primers containing EcoRI and BamHI restriction enzyme digest sites. Following agarose gel PCR product purification (QIAquick gel Extraction Kit, Qiagen), WT RNase H1-V5 CDS was subcloned into LVR-1003-MCS-PGK-BSD with EcoRI and BamHI restriction enzymes to generate lentiviral WT RNase H1-V5 plasmid. A second-generation lentiviral system was used to generate WT RNase H1-V5 expressing lentivirus. Briefly, VSV-G envelop-expressing, psPAX2 packaging (addgene plasmids #12,260 and #12,259; a generous gift from D. Trono), and lentiviral WT RNase H1-V5 transfer plasmids were transfected into HEK293T cells using X-treme Gene HP DNA Transfection Reagent (Millipore, Sigma). Twenty-four hrs post-transfection, HEK293T culture medium was replaced with DMEM supplemented with 10% FBS and pen/strep antibiotics (Invitrogen). Twenty-four hrs later, the viral supernatant was collected and concentrated at 26,000 rpm for 1 h and 45 min in an Optima L-100 XP Ultracentrifuge (Beckman Coulter). Thereafter, lentivirus was snap-frozen in liquid nitrogen and stored in -80 °C. Immortalized UtSMCs were used to generate a stable line expressing WT RNase H1-V5. Briefly, 70% confluent UtSMCs were transduced with WT RNase H1-V5 lentivirus supplemented with 2 μg/mL of polybrene. Twenty-four hrs post-transduction, culture media was replaced with the culture media containing 2 μg/mL of Blasticidin (BSD) selectable marker. WT RNase H1-V5 (hereafter simply RNaseH)-expressing cells were selected with BSD for one week. Expression of WT RNase H1-V5 was validated by immunoblot analysis with V5-specific antibody.

### Immunofluorescent staining

UtSM and UtSM-RNAseH overexpressing cells were plated in PerkinElmer cell carrier imaging 96- well plates at a density of 4000–5000 cells/well. Cells were treated with vehicle (DMSO) control, CCT254515 (100 nm), or Camptothecin (10 mM) for the times indicated in each individual experiment. Cells were fixed with 4% paraformaldehyde and processed for immunofluorescent detection as described earlier^55^ and imaged in a single focal plane at × 40 magnification using an Operetta™ imaging system. Using Columbus™ (PerkinElmer) software, the protein intensity in the cytoplasm and nucleus were quantified. The nucleus was defined by DAPI staining (Thermo Fisher Scientific) and processed for further analysis .

### MED12 mutation analysis

Total DNA was isolated form the myometrial and fibroid tissue using QuickExtract DNA Extraction Solution (Lucigen), according to manufacturer’s instruction. Following total DNA extraction, genomic region containing UF-linked MED12 mutations was PCR amplified with the following primers: MED12-F: GCCCTTTCACCTTGTTCCTT and MED12-R: TGTCCCTATAAGTCTTCCCAACC. PCR product was gel purified using QIAquick Gel Extraction Kit (Qiagen), according to manufacturer’s instructions, and Sanger Sequenced by Genewiz, (NJ, USA).

### Primary cell isolation and culture

Primary cells were isolated as described^56^. Briefly, MM and UF tissues were diced manually into small pieces of < 1 mm^3^ which were then incubated overnight (16-18 h) in DMEM/F12 (GIBCO) containing 0.2% (wt/vol) collagenase (Wako), 0.05% DNase I (Invitrogen), 1% antibiotic–antimycotic mixture (Invitrogen), 10% FBS and 10 mM Hepes buffer solution (Invitrogen) at 37 °C on a shaker. After shaking, the digested tissue was filtered through a sterile 100-μm polyethylene mesh filter to remove undigested tissues, and again filtered through a 40-μm cell strainer (BD–Falcon). The filtrates were treated with ACK lysis solution for 10 min at room temperature, centrifuged and washed with 1X HBSS to remove red blood cells. Live cells were counted and seeded in 10 cm dishes (250,000cells/dish) with DMEM/F12 (GIBCO) with 10% fetal bovine serum and 1% antibiotic–antimycotic (Invitrogen) at 5% CO2. Primary cell cultures were processed within 3 days of plating for DNA fiber analysis.

### DNA fiber analysis

Primary MM and UF cells were labeled with 50 μM IdU (Sigma-Aldrich I-7125) followed by 100 μM CldU (Sigma-Aldrich C-6891) for 30 min each as described previously^57^. UtSM and UtSM-RNaseH expressing cells were treated with 100 nM CCT254515 for 2 h in between Idu and Cldu treatments. Briefly, ~ 300,000 cells were embedded in agarose and DNA was prepared then combed onto silanized coverslips using the FiberComb® Molecular Combing System (Genomic Vision). Following combing, coverslips were baked for 2 h at 65 °C, dehydrated in ethanol (70%-90%-100%, 3 min each), then denatured with 0.5 M NaOH + 1 M NaCl for 8 min at room temperature. Coverslips were neutralized with PBS (3 min wash, 3 times), subjected to a graded ethanol series as described above and air-dried. Combed DNA was blocked with BlockAid Blocking Solution (Invitrogen B10710), followed by immunostaining with antibodies specific for IdU (mouse anti-BrdU, BD Biosciences 347,580) and CldU (rat, anti-BrdU, Abcam ab220074) for 1 h at 37 °C, washed with PBS-T, and probed with secondary antibodies (anti-mouse, Cy3, SIGMA C2181 and anti-rat, AF488, Invitrogen A11006) for 45 min at 37 °C. Single-stranded DNA was counterstained with anti-ssDNA mouse antibody (DSHB University of Iowa) for 2 h at 37 °C, followed by anti-mouse BV480 (Jackson Immuno Research 115–685-166) for 45 min at 37 °C. Coverslips were washed in PBS, subjected to a graded ethanol series, air-dried and then mounted. Images were obtained using a 40 × oil objective on a confocal microscope Nikon Swept field. The number of stalled forks, new forks, bidirectional forks, and their lengths were measured using Image J. To calculate fork velocity, the following equation was used to convert fork length from μm to kb/min: length μm × 2.59/labeling time in min = fork velocity kb/min.

### Cell cycle and proliferation analyses

For cell cycle analyses, UtSM and UtSM-RNaseH expressing cells (3 × 10^5^) were seeded in 15-cm plates and cultured for 24 h before the addition of DMSO or CCT254515 (100 nM) for an additional 72 h. Cells were trypsinized with 0.05% Trypsin–EDTA, centrifuged for 10 min at 1000 RPM, 4 °C and washed with 1 mL 1 × PBS three times. Thereafter, cells (1 × 10^6^) were fixed with 70% ethanol at 4 °C overnight. Fixed cells were centrifuged for 5 min at 2800 RPM at 4 °C. After ethanol removal, the cells were treated with 40 µg/mL of Propidium Iodide and 0.1 µg/mL of RNaseA in 1 × PBS and incubated for 30 min at 37 °C. Stained cells were filtered through a cell strainer and incubated on ice before FACS analysis. Cell cycle analysis was performed on BD FACSCalibur and data analysed using Flowjo software. For proliferation assays, UtSM and UtSM-RNaseH expressing cells (1 × 10^4^) were seeded in 6-well plates and cultured for 24 h before addition of DMSO or CCT254515 (100 nM). Cells were harvested at 48, 96, and 144 h post treatment, stained with 0.4% trypan blue, and manually counted on a hemocytometer. All experiments were performed in triplicate.

### Immunoblot analysis

Validation of target engagement and kinetics of Mediator kinase inhibition by CCT251545 in UtSMCs was performed by immunoblot analysis of STAT1 as described previously^33^ using antibodies specific for total (SC-464; Santa Cruz Biotechnology) and phosphorylated (Ser727) (#9177; Cell Signaling Technology) STAT1.

### Statistical analysis

Statistical testing was performed using Graph Pad Prism 9. One way ANOVA followed by post Hoc test was used to calculate significance. For the other experiments, a two-sided unpaired Student’s t-test was calculated. Significance was assumed where *p*-values ≤ 0.05. Asterisks represent significance in the following way: ****p ≤ 0.0001, ***p ≤ 0.001; **p ≤ 0.01; *p ≤ 0.05.

All methods were carried out in accordance with relevant guidelines and regulations.

## Supplementary Information

Supplementary Information 1.

Supplementary Information 2.

## Data Availability

All data generated or analyzed during this study are included in this published article (and its Supplementary Information files).
