# Supplementary material for: Aberrant R-loop-induced replication stress in MED12-mutant uterine fibroids
Source: Sci Rep. 2022 Apr 13;12:6169. doi: 10.1038/s41598-022-10188-x (PMC9008039; doi:10.1038/s41598-022-10188-x)
Supplement: Supplementary file 1 — Supplementary Information 1. [file 41598_2022_10188_MOESM1_ESM.pdf]

Supplemental Material

Includes:
Supplemental Figures and Legends

Aberrant R-loop induced replication stress in MED12-mutant uterine fibroids

Sribalasubashini Muralimanoharan[1], Ross Shamby[1], Nicholas Stansbury[2], Robert Schenken[2], Barbara de la Pena Avalos[3], Samin Javanmardi[1], Eloise Dray[3], Patrick Sung[3] & Thomas G. Boyer[1]*

[1]Department of Molecular Medicine, UT Health San Antonio, San Antonio, TX USA
[2]Department of Obstetrics and Gynecology, UT Health San Antonio, San Antonio, TX USA
[3]Department of Biochemistry and Structural Biology, UT Health San Antonio, San Antonio, TX USA

*To whom correspondence should be addressed: boyer@uthscsa.edu
Department of Molecular Medicine
UT Health San Antonio
8210 Floyd Curl Drive
Mail Code 8257, STRF
San Antonio, TX 78229-3900
210-562-4151

Supplemental Figure S1

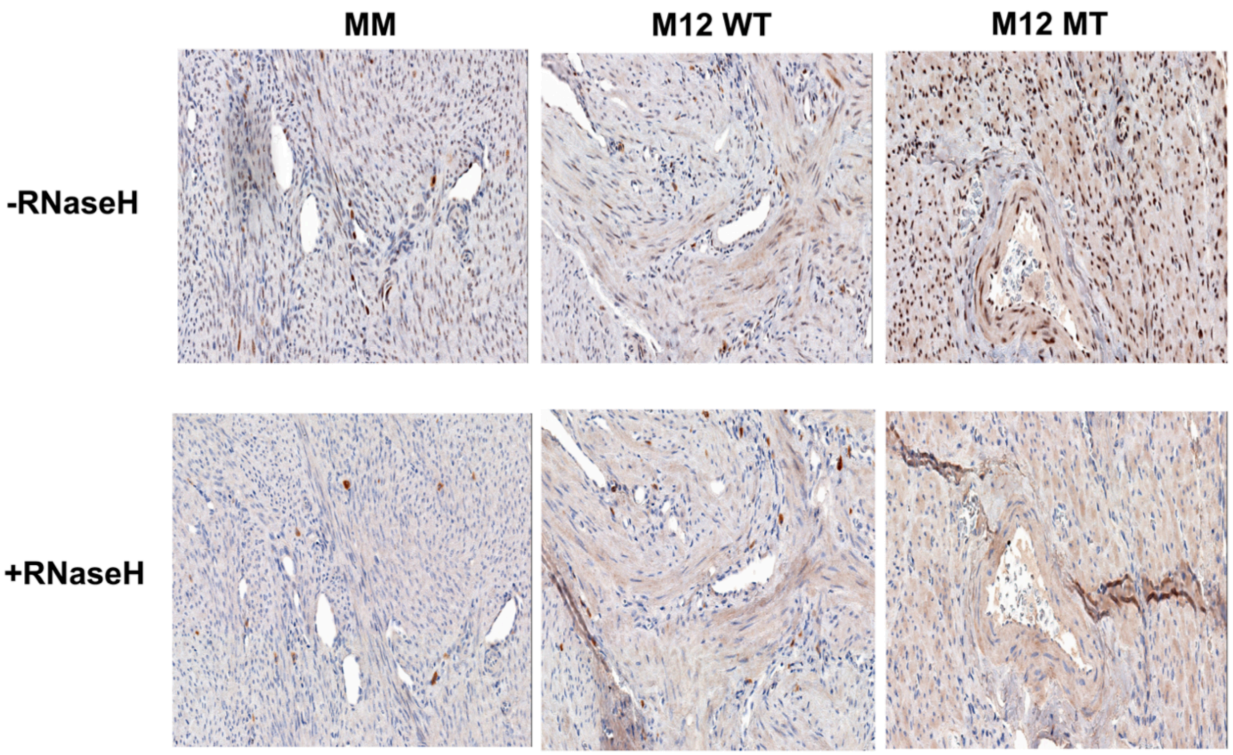

Figure S1. Antibody S9.6 specificity test for RNA-DNA hybrids on TMAs. Representative TMA IHC staining with R-loop specific S9.6 antibody performed without (-) or with (+) RNaseH pretreatment. Shown are images from 1 patient-matched tissue sample set (of 10 total arrayed tissue sample sets tested). Each sample set includes tissue from MM, as well as one MED12-wild-type (WT) and one MED12-mutant (MT) UF all derived from the same patient uterus. TMAs were treated without or with 1U RNaseH for 60 min before incubation with S9.6 antibody and further IHC processing as described in Material and Methods. All 10 arrayed tissue sample sets showed abolishment of S9.6 signal by prior treatment with RNaseH.

Supplemental Figure S2

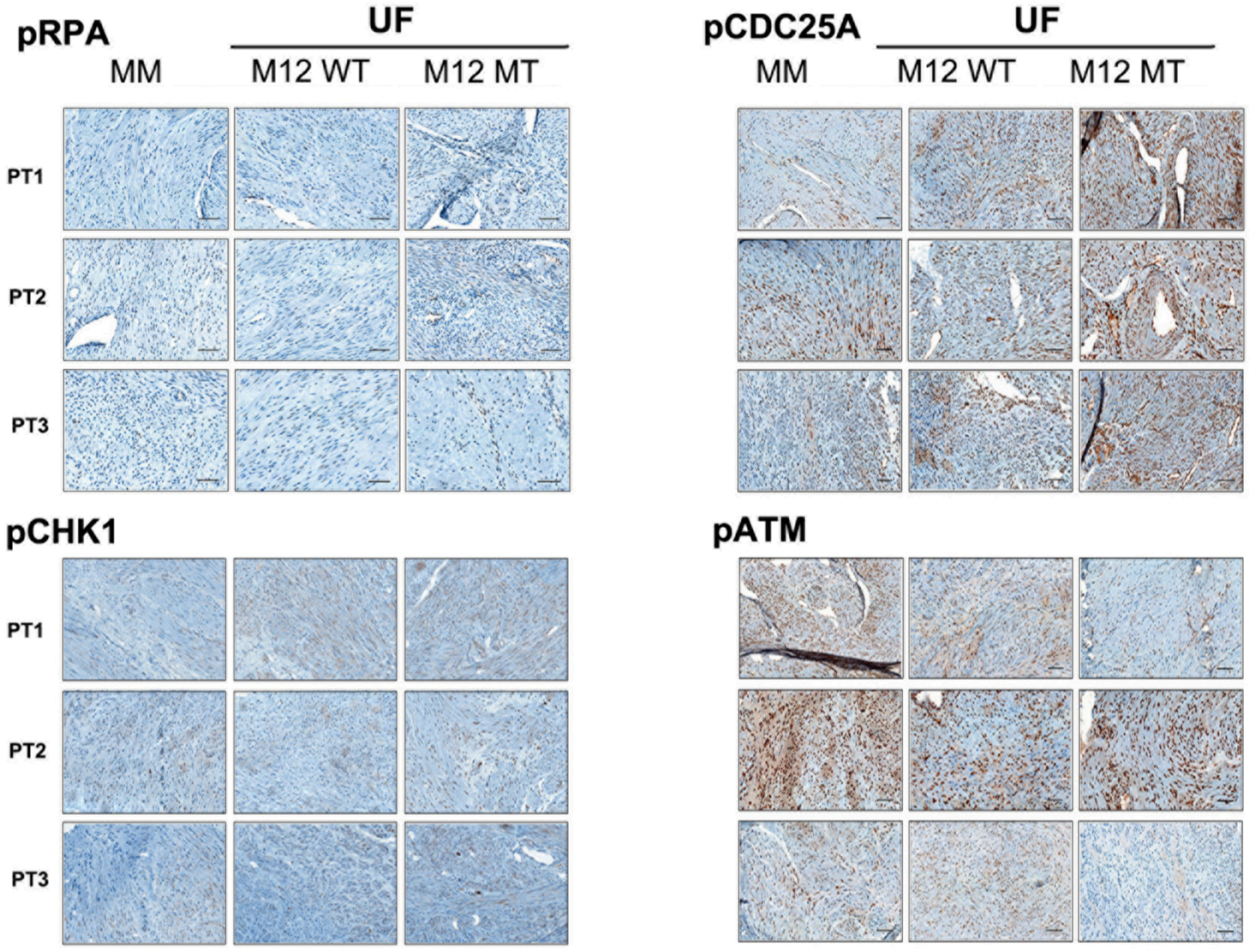

Figure S2: Analysis of replication stress signaling markers in patient-matched MM and UF tissues. Representative IHC staining with antibodies specific for pRPA (Ser33), pCHK1 (Ser317), pCDC25A (Ser124), and pATM (s1981). Shown are images from 3 patient-matched tissue sample sets (PT1-PT3), each set comprising MM, MED12-wild-type (M12 WT) and MED12-mutant (M12 MT) UF tissue. Data were quantified as described from IHC analysis of 10 patient-matched tissue sample sets and quantified data are shown in Figure 1.

Supplemental Figure S3

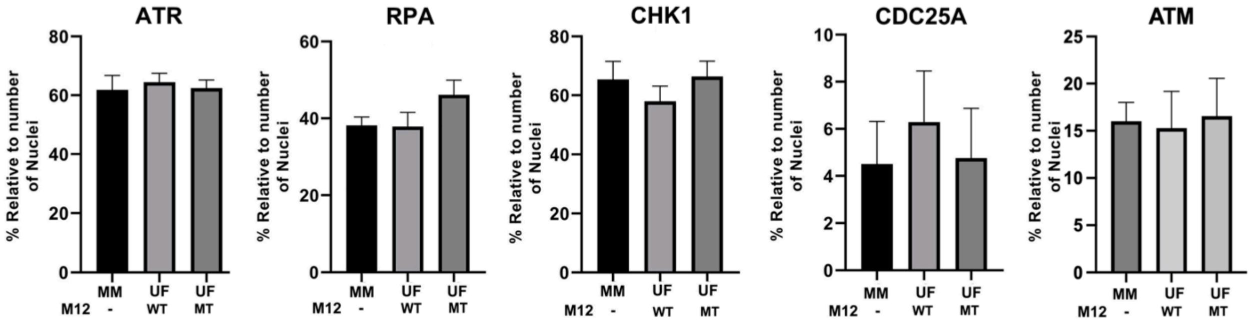

Figure S3. Bulk expression levels of replication stress signaling proteins and ATM are not elevated in MED12-mutant UFs compared to MM and MED12-WT UFs. Quantified expression of bulk replication stress signaling proteins ATR, RPA, CHK1, CDC25A and ATM by IHC in MM and UF tissues. Data were quantified from IHC analysis of 10 patient-matched tissue sample sets, each set comprising MM as well as one MED12-WT (WT) UF and one MED12-mutant (MT) UF all derived from the same patient uterus. Stained sections were scanned using an Aperio ScanScope® CS system, and individual nuclei as well as nuclear and cytoplasmic marker localization were identified and quantified using Aperio ImageScope software. Signals were normalized to the number of nuclei in each section. Statistical significance was calculated using One-Way ANOVA followed Tukey's Post hoc test. No statistically significant differences in quantified expression of bulk replication stress signaling proteins or ATM were observed between MM or UF tissues.

Supplemental Figure S4

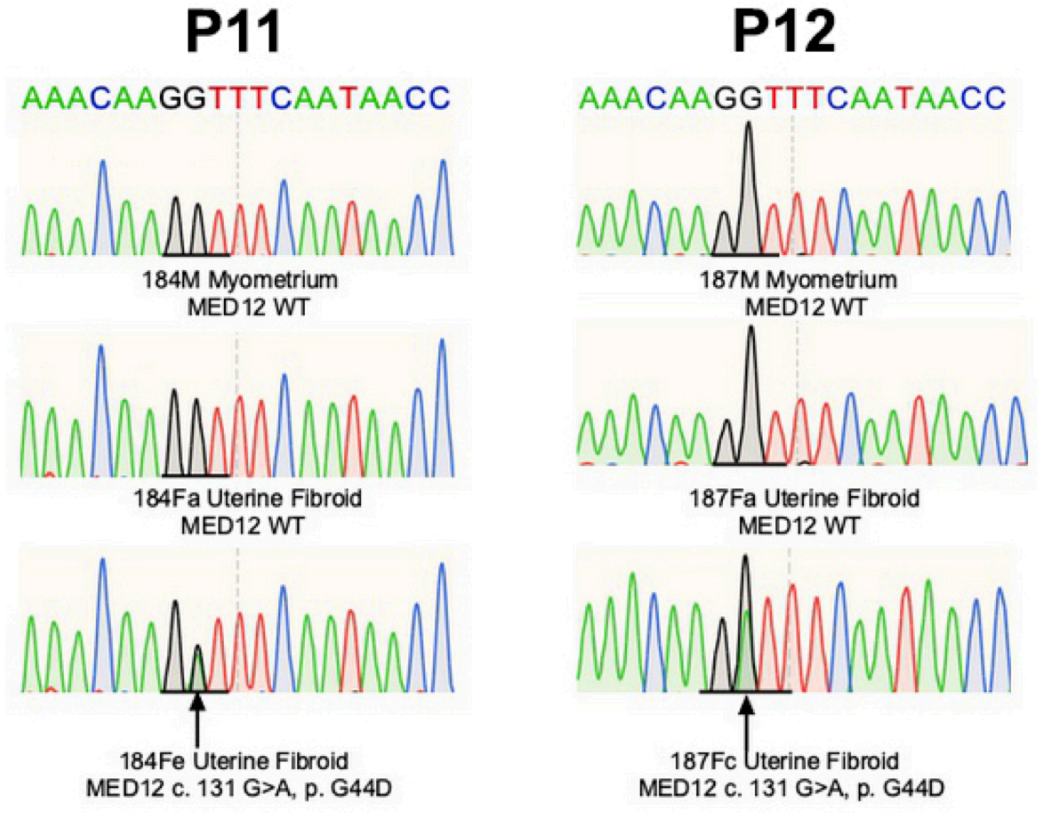

Figure S4. MED12 mutation status in patient-derived UF tissues used for DNA fiber analysis. Genomic DNA extracted from 2 different patient-matched tissue sample sets, each set comprising MM and UF tissues from the same patient uterus, was PCR-amplified using primers flanking *MED12* exon 2 and subjected to Sanger sequencing. Shown are sequencing chromatograms from Patient 11 (P11) and Patient 12 (P12), which were confirmed to harbor one MED12-WT UF and one MED12-mutant UF each. The MED12-mutant UF from P11 corresponded to c.131G>A, p.G44D. The MED12-mutant UF from P12 corresponded to c.131C>A, p.G44D.

Supplemental Figure S5

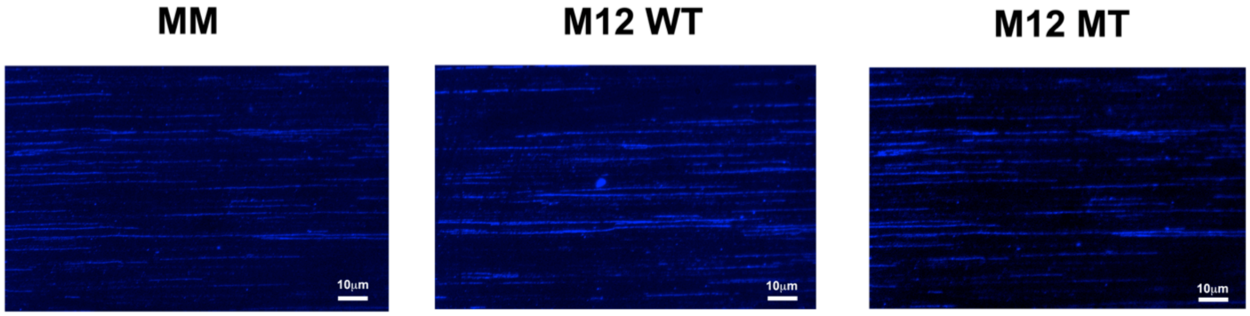

Figure S5. Representative images of combed DNA fibers from primary MM and UF cells stained for ssDNA. Primary MM and UF cells were pulse labeled with thymidine analogues IdU and CldU. Naked DNA from pulse labeled MM and UF cells was prepared and then combed in triplicate onto silanized coverslips using the FiberComb® Molecular Combing System (Genomic Vision). One set of cover slips shown here was processed by immunostaining with mouse anti-ssDNA and anti-mouse BV480 as described in Materials and Methods. A replicate set of cover slips was processed for immunostaining using analog-specific antibodies as shown in Figure 2. The MED12 mutation status of UF cells [MED12 WT (M12 WT) or MED12-mutant (M12 MT)] is indicated.

Supplemental Figure S6

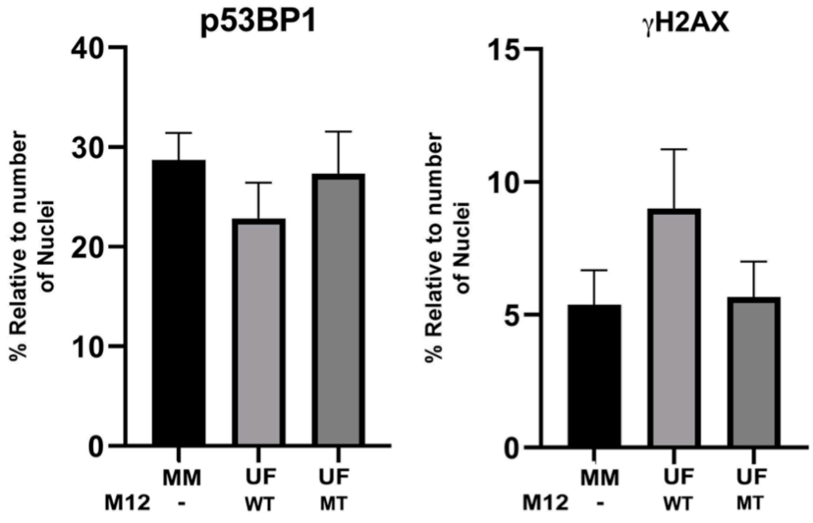

Figure S6. Activated DNA damage markers are not increased in MED12-mutant UFs compared to MM and MED12-WT UFs. Quantified expression of activated DSB marker proteins p53BP1 (Ser25) and γH2AX (Ser139) by IHC in MM and UF tissues. Data were quantified from IHC analysis of 10 patient-matched tissue sample sets, each set comprising MM as well as one MED12-WT (WT) UF and one MED12-mutant (MT) UF all derived from the same patient uterus. Stained sections were scanned using an Aperio ScanScope® CS system, and individual nuclei as well as nuclear and cytoplasmic marker localization were identified and quantified using Aperio ImageScope software. Signals were normalized to the number of nuclei in each section. Statistical significance was calculated using One-Way ANOVA followed Tukey's Post hoc test. No statistically significant differences in quantified expression of DSB marker proteins were observed between MM or UF tissues.

Supplemental Figure S7

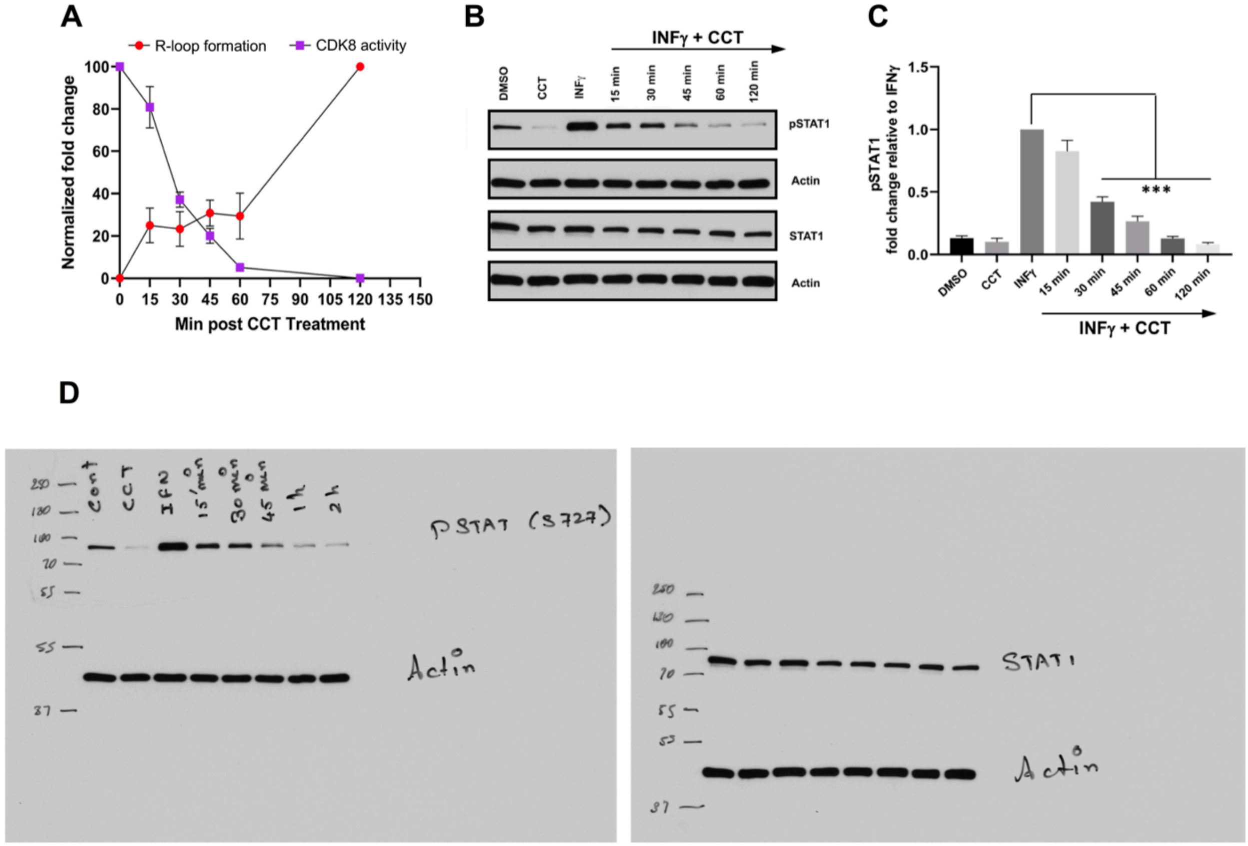

Figure S7. Validation and temporal kinetics of target engagement by Mediator kinase inhibitor CCT251545 in UtSMCs. (**A**) Temporal kinetics of Mediator kinase inhibition and R-loop formation in UtSMCs. UtSMCs treated with DMSO or CCT251545 (CCT; 100nM) were processed at the indicated time points for analysis of R-loop formation by immunocytochemical staining with S9.6 antibody or Mediator kinase activity by immunoblot analysis for pSTAT1^{S747}. To monitor pSTAT1^{S747}, cells were pretreated for 45 min with IFNγ (10ng/ml) and the normalized level of pSTAT1^{S747} to bulk STAT1 was quantified (see panels **B** and **C**). Fold-change in Mediator kinase activity or R-loop formation at each time point was plotted relative to the level in DMSO-treated cells. (**B** and **C**) UtSMCs treated with DMSO or CCT251545 (CCT: 100nM) as indicated along with interferon gamma (INFγ; 10ng/ml) were processed for immunoblot analysis using antibodies specific for phosphorylated STAT1^{S727} (pSTAT1), bulk STAT1, and β-ACTIN, the latter of which served as an internal loading control. The level of phosphorylated STAT1 under each condition was determined by quantification of immunoblot signals and normalization of the pSTAT1 signal to that of both bulk STAT1 and β-ACTIN. Panel B shows a representative immunoblot. Data from three independent immunoblot experiments were used to derive quantitative data in panel C. Statistical significance was calculated using One-Way ANOVA followed Tukey's Post hoc test, ***$p<0.001$ compared to INFγ alone. (**D**) Original immunoblots from which cropped sections in panel B were derived.

Supplemental Figure S8

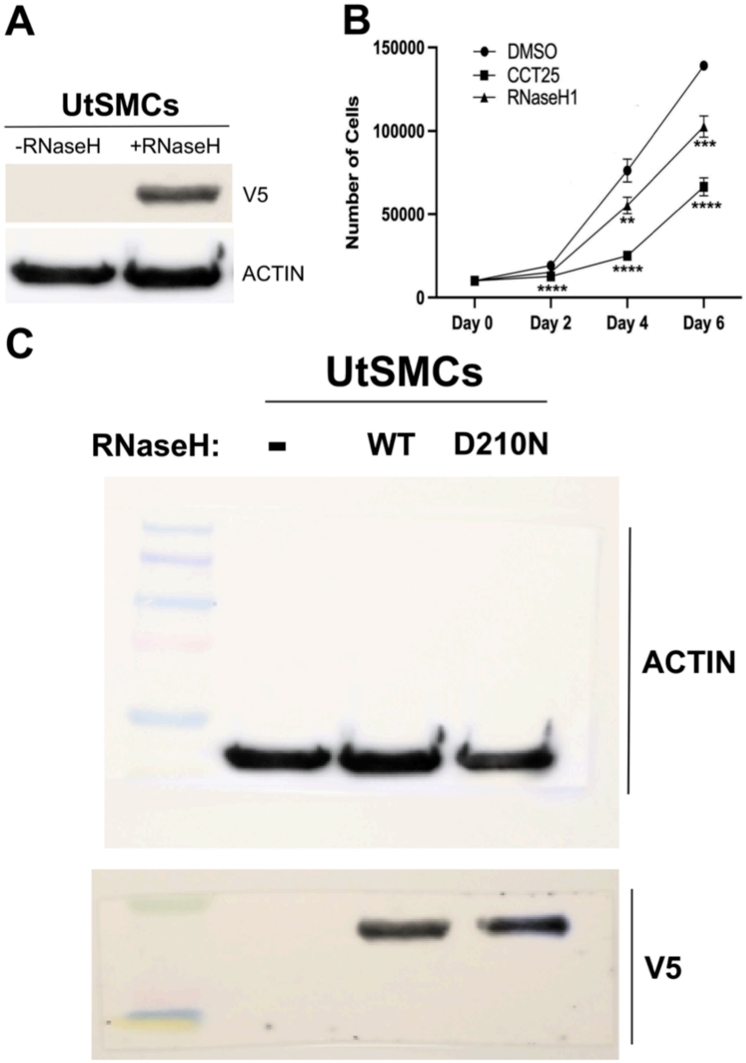

Figure S8. Mediator kinase inhibition leads to a proliferative defect in UtSMCs that is reversed by overexpression of RNaseH. (**A**) Validation of RNaseH overexpression in UtSMCs. Whole cell lysates from parental (-RNase H) and V5 epitope-tagged RNaseH overexpressing (+RNase H) cell lines were processed in parallel by immunoblot analysis using antibodies specific for the V5 epitope or β-ACTIN (internal loading control). (**B**) CCT251545 treatment triggers an R-loop-dependent proliferation defect in UtSMCs. Parental or RNaseH overexpressing UtSMCs (1 x 10^4 cells) were seeded in 6-well plates and cultured for 24 hours before addition of DMSO or CCT254515 (CCT; 100nM). Thereafter, cells were harvested at 2, 4, and 6 days post treatment for live cell counting. Data were derived from 3 independent experiments, each performed in triplicate. Statistical significance was calculated using One-Way ANOVA followed by Tukey's Post hoc test, ****p ≤ 0.0001, ***p<0.001; **p<0.01 compared to DMSO. (**C**) Original immunoblots from which cropped sections in panel A were derived. Lanes labelled -RNaseH and +RNaseH in panel A correspond to RNaseH (-) and RNAseH (WT) lanes in panel C. D210N corresponds to a catalytically deficient RNaseH derivative that was not used in these studies.

Supplemental Figure S9

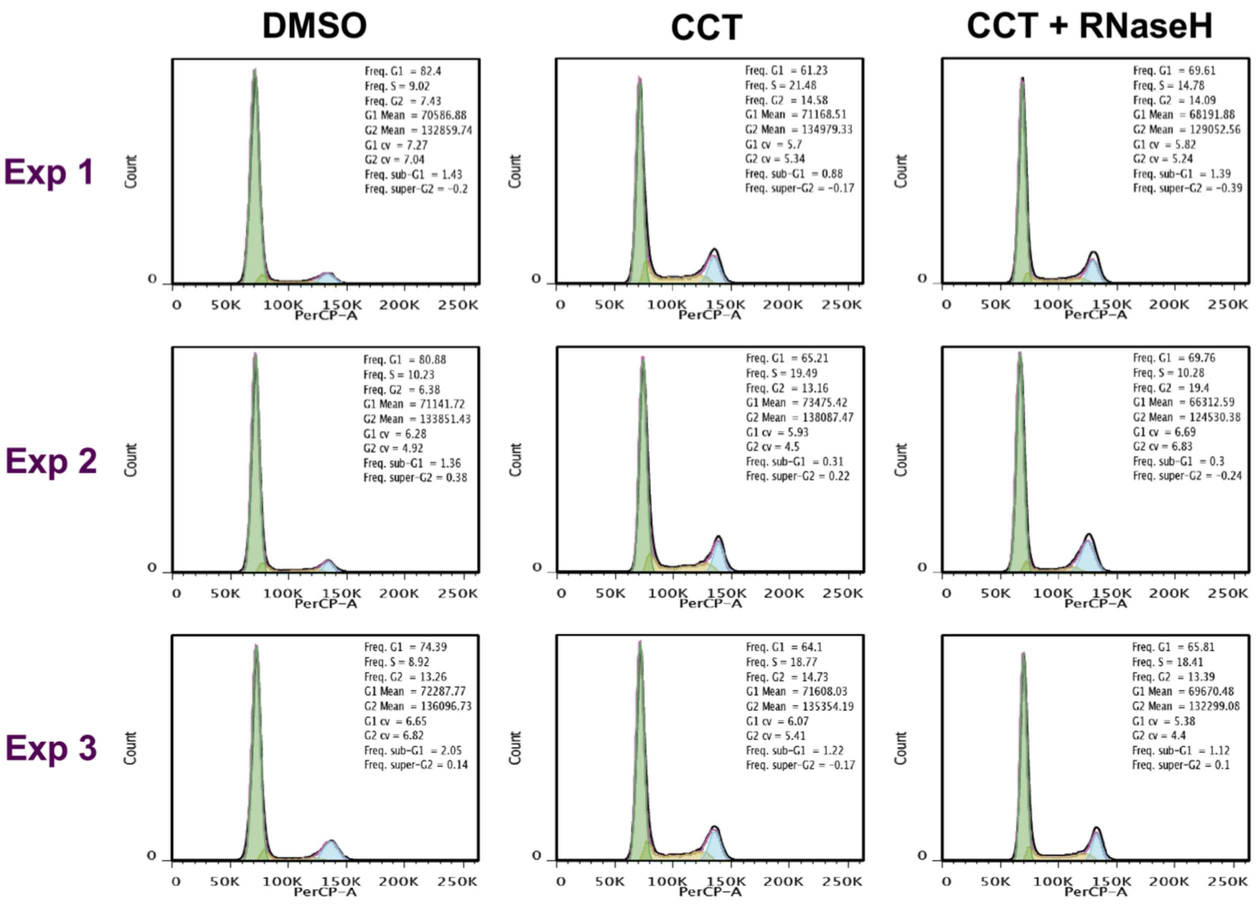

Figure S9. Mediator kinase inhibition triggers an S-phase cell cycle delay in UtSMCs. Unsynchronized parental or RNaseH overexpressing (+ RNaseH) UtSMCs treated with DMSO or CCT251545 (CCT; 100nM) for 3 days were stained with propidium iodide and analyzed by flow cytometry. FACs histograms from 3 independent experiments are shown. The percentage of cells in each cell cycle phase based on aggregate data is plotted in Figure 4E.
