# Supplementary material for: Aberrant R-loop-induced replication stress in MED12-mutant uterine fibroids
Source: Sci Rep. 2022 Apr 13;12:6169. doi: 10.1038/s41598-022-10188-x (PMC9008039; doi:10.1038/s41598-022-10188-x)
Supplement: Supplementary file 2 — Supplementary Information 2. [file 41598_2022_10188_MOESM2_ESM.pdf]

Table S1

Table S1: Summary of clinical demographics data for patient samples

Sample number	Ethnicity	Age	Solitary/Multiple fibroids	Sample ID	UF M12 Status (WT, MT)
1	Caucasian	44	multiple	89	Fc, Fb
2	Hispanic	47	multiple	93	Fa, Fb
3	Hispanic	42	multiple	104	Fa, Fb
4	Hispanic	42	multiple	110	Fb, Fa
5	Hispanic	48	multiple	118	Fa, Fe
6	Hispanic	60	multiple	121	Fb, Fa
7	Hispanic	48	multiple	125	Fa, Fb
8	Hispanic	44	multiple	88	Fa, Fb
9	Hispanic	NR	multiple	99	Fb, Fc
10	Hispanic	35	multiple	119	Fa, Fc
11	Hispanic	49	multiple	184	Fa, Fe
12	Hispanic	51	multiple	187	Fa, Fc

NR-not recorded.

Patient samples 1-10, including MM and UF MED12 mutation status, have been described previously by us (15). Information related to these patient samples can be accessed in reference (15) by the corresponding sample ID. For Patients 11 and 12, MM and UF MED12 mutation status are shown in Supplemental Figure S3.
